# Genetic depletion of early autophagy protein ATG13 impairs mitochondrial energy metabolism, augments oxidative stress, induces the polarization of macrophages to M1 inflammatory mode, and compromises myelin integrity in skeletal muscle

**DOI:** 10.21203/rs.3.rs-7189456/v1

**Published:** 2025-08-06

**Authors:** Mubaraq A Toriola, Emma Timlin, Sarojini Bulbule, Amy Reyes, Omolola Mary Adedeji, C Gunnar Gottschalk, Animesh Barua, Leggy A Arnold, Avik Roy

**Affiliations:** University of Wisconsin–Milwaukee; University of Wisconsin–Milwaukee; Simmaron Research INC; Simmaron Research INC; University of Wisconsin–Milwaukee; Simmaron Research INC; Simmaron Research INC; University of Wisconsin–Milwaukee; Simmaron Research INC

## Abstract

M1 macrophage activation is crucial in chronic inflammatory diseases, yet its molecular mechanism is unclear. Our study shows that hemizygous deletion of early autophagy gene *atg13* (Tg^+/−^ATG13) disrupts cellular autophagy, hinders mitochondrial oxidative metabolism, increases reactive oxygen species (ROS) in splenic macrophages, leading to its M1 polarization. Reduced macroautophagy markers WDFY3 and LC3, flow-cytometric analysis of M1/M2 markers (CD40, CD86, CD115, CD163, and CD206), deficit of oxygen metabolism evaluated by ROS-sensor dye DCFDA, and seahorse oxygen consumption studies revealed that atg13 gene ablation impairs mitochondrial function triggering M1 polarization. Additionally, redox imbalance may impair Sirtuin-1 activity via nitrosylation, increasing the level of acetylated p65 in macrophages contributing to the inflammatory response in M1Mφ. Additionally, the ablation of the atg13 gene resulted in the increased infiltration of M1Mφ in muscle vasculature, deterioration of myelin integrity in nerve bundles, and a reduction in muscle strength following treadmill exercise. These findings underscore the significance of ATG13 in post-exertional malaise (PEM).

## Introduction

Autophagy[1] is a mechanism of cellular quality control metabolism, by which defective cellular proteins[2] and depolarized mitochondria[3] are enclosed in a bilayer vesicle known as autophagosome followed by its targeting and fusion to lysosome for the degradation (). The formation of the autophagosome[4] is programmed by the concerted action of a class of proteins termed as autophagy related proteins or ATGs[5]. Over 36 ATG proteins[6], primarily identified from yeast autophagy mutants[7], drive the autophagic process[8]. ATG13 primarily regulates autophagosome formation in the context of starvation-induced condition[9], however it also plays a critical role in the starvation-independent or constitutive autophagy[10] as well. The ATG9/2/18 transmembrane complex promotes phagophore expansion by shuttling membrane from sources like the endoplasmic reticulum, trans-Golgi network, and mitochondria[11]. The ATG7/ATG3/Atg8 and ATG7/ATG10/ATG12 ubiquitin-like conjugation systems are involved in enclosing autophagosomal membranes [12].

Previous literatures[13, 14] demonstrated that the loss of function of the atg13 gene in a Tg-ATG13 knock-out first mouse model impaired autophagy, which led to the increased infiltration of inflamed mononuclear cells into the vasculature of muscle parenchyma. This caused a demyelinating response in muscle-serving nerves, resulting in chronic muscle fatigue. Further analysis revealed that these mononuclear cells are CD40^+ve^ and iNOS^+ve^ inflammatory macrophage (Mφ) cells. However, the mechanism by which ATG13 ablation triggers the inflammation in Mφ cells is not known.

The inflammatory and chronic demyelinating pathologies due to atg13 ablation could be relevant to the pathogenesis of Myalgic encephalomyelitis or chronic fatigue syndrome (ME/CFS)[14] and therefore may shed light in the molecular mechanism in post-exertional malaise (PEM). PEM is a key symptom of ME/CFS[15, 16] that involves significant muscle fatigue, pain, and cognitive deficits following physical or emotional exertion. Recent literatures indicate that glucose metabolism deficits[17], reduced mitochondrial oxygen consumption [18], defective energy metabolism[19], and immune cell depletion [20] may contribute to the PEM, though the molecular mechanism remains unclear. In our current study, a comprehensive flow cytometry analyses highlights that Tg^+/−ATG13^ mice with significantly depleted atg13 gene induces the polarization of M1Mφ in spleen. Mitochondrial stress studies such as oxygen consumption analyses and ROS production study further indicated that these M1Mφ cells are deficient of oxygen consumption and energy metabolism, which may trigger the inflammatory response causing the enhanced infiltration in muscle vasculature and potential demyelination in muscle-serving nerves. Collectively, our present study delineates the molecular role of the atg13 gene in promoting muscle inflammation via activation of M1Mφ cells.

## Materials and Methods

### Reagents, media, buffers, kits, and antibodies:

TBS (10X) (Ref#28358), 20X PBS-Tween (Ref#28352), NuPAGE^™^ MOPS SDS Running buffer (Ref#NP0001), 20X NuPAGE^™^ Transfer buffer (Ref# NP00061), 20X TE buffer (Cat#TE03), and 5X TBE (Cat#J63487.K3), NuPAGE^™^ 4–12% Bis-Tris Gel (Ref# NP0322BOX), Nitrocellulose membrane (0.45 μm; Ref# 88025) were purchased from Thermo Fisher Scientific (MA, USA). DMEM (Cat # 11995–065), STEMpro^®^ Accutase^®^ (Ref# A11105–01) were purchased from Gibco, Thermo Fisher Scientific. Intercept TBS blocking buffer (Part# 927–60001), antibody diluent (Part# 9297–95001), IRDye^™^ 680 Donkey anti-rabbit (Part# 926–68073), IRDye^™^ 680 Donkey anti-mouse (Part# 926–68072), IRDye^™^ 680 Goat anti-rabbit (Part# 926–68071), IRDye^™^ 680 Goat anti-mouse (Part# 926–68070), nitrocellulose membrane (Part# 926–31090) were purchased from Licor Biosciences (NE,USA). TritonX (0.05%) -conjugated eBioscience^™^ Flow Cytometry Staining Buffer (Ref# 00–4222-57; ThermoFisher INC) was used to wash, neutralize non-specific binding and achieve the optimum permeabilization for intracellular staining. All primary antibodies were listed below with catalogue number, vendor, and application.

### Generation of Tg^+/− ATG13^ mice and phenotype assessment.

The ATG13 repressor mouse strain used for this research project, C57BL/6N-Atm1Brd Atg13tm2a(EUCOMM)Hmgu/BayMmucd, RRID:MMRRC_041527-UCD, was obtained from the Mutant Mouse Resource and Research Center (MMRRC) at University of California at Davis, an NIH-funded strain repository, and was donated to the MMRRC by Arthur Beaudet, M.D., Baylor College of Medicine. Mice were generated at the Baylor College of Medicine as part of the Baylor College of Medicine, Sanger Institute, and MRC Harwell (BaSH) Consortium for the NIH Common Fund program for Knockout Mouse Production and Cryopreservation (1U42RR033192–01) and Knockout Mouse Phenotyping (1U54HG006348–01). After generation of the strain, these ATG13 repressor mice were transported, housed, and bred at the CRC animal facility of UWM according to the approved IACUC protocol: **22–23 #20**. To selectively knock-out atg13 gene these mice were bred with B6.C-Tg(CMV-cre)1Cgn/J, the mice that express cre recombinase enzyme and designed to delete loxP-flanked *atg13* gene preferentially in males (via X-linked gene transmission). After 5 generations of breeding and genotyping, we were unable to receive any homozygous *atg13* knock-out offsprings, possibly due to the augmented lethality during gestational period. However, we have successfully generated and accumulated sufficient hemizygous male mice after breeding for 7 generations. Although, there is no apparent developmental deficit and growth disorder, these mice display slower movement, anxiety, and poor motor performance once subjected to treadmill stressor. Male mice gain body weight faster than wild-type mice starting from 6 months of their age with significant increase of abdominal fat. Following Primer-pairs (postCre: product length =797 bp) were used for identifying the Tg^+/ATG13^ genotype. Primer#1: 5’---GCTACCATTACCAGTTGGTCTGGTGTC-−−3’ and Primer #5: 5’---CACCATCTGTAATGGGATCCAAAGGC-−−3’. The preCre bands appear at 499 bp.

### Preparation of Splenocytes and enrichment of macrophages (Mφs).

To isolate Mφ-enriched splenocytes, spleen tissue was harvested and homogenized in 5 mL of sterile HBSS for 2 minutes. It was centrifuged at 1200 rpm for 5 minutes, then digested with 1 mL of accutase enzyme at 37°C for 5 minutes. The digestion was neutralized with 1X HBSS, followed by centrifugation and resuspension in complete DMEM/F12 media. The pellet was disintegrated with pipetting, seeded in a flask at 37°C for 10 minutes, and the media containing suspended cells was removed. The adherent cells were primarily Mφs with some monocytes. The cells were carefully harvested in serum-free DMEM/F12 medium for subsequent immunoassays without any further addition of M1 or M2-specific growth factors. This condition was essential for our study to minimize potential confounding variables arising from media composition.

### Flowcytometry analysis.

Enriched Mφs were isolated from spleen of 10–12 weeks ‘old male NTg and Tg^+/− ATG13^ mice as described above followed by counting cell numbers in Countess^™^ 3 automated cell counter (ThermoFisher Scientific, MA, USA). An BD Accuri^™^ C6 Plus (BD Biosciences, San Jose, CA) flow cytometer was used, which is equipped with a blue (488nm) and red (640nm) laser, two light scatter detectors and four fluorescence detectors with optical filters (FL1= 533/30, FL2= 585/40, FL3= 670 LP / 610/20 and FL4= 675/25). At least 10,000 total events were calculated for the analyses once the compensation were performed using CompBead Fluorescence matrix according to manufacturer’s recommendations. Briefly, cells were suspended in eBioscience^™^ Flow Cytometry Staining Buffer (Ref# 00–4222-57; ThermoFisher INC) and then stained with FITC-, PE-, or APC-conjugated antibodies for 30 min at room temperature, washed (3X), and then subjected to analysis in flow cytometer. Results were analyzed using FlowJo V10.10.0 software as per standard guidelines.

### Immunofluorescence (IF) analysis:

IF analysis was performed as described elsewhere[21]. Muscle tissue were mounted in a paraffin-embedded blocks and then sectioned at a thickness of four microns in our Leitz 1512 manual microtome. Subsequently, the sections were sequentially treated with xylene, then 100%, 70%, 50%, and 20% ethanol, and rehydrated in water following standard IHC protocol. Blocking was done using 2% BSA buffer, followed by overnight incubation with primary antibodies at room temperature. Tissue sections were washed with 1X PBST, then incubated with a biotinylated secondary antibody. For DAB staining, biotin-conjugated 2° antibodies were used per the dual IHC kit (Abcam; Cat # ab210059). For immunofluorescence, FITC- and TRITC-conjugated secondary antibodies (Jackson Immuno Research) were applied. Slides were deparaffinized, underwent antigen retrieval in citrate buffer (pH 5.5), blocked with 2% horse serum, treated with primary antibodies (1:100–1:250), washed with 1X TBST, incubated with labeled antibodies, washed again (DAPI included in final wash at a dilution of 1:10,000), and cover slipped. Imaging was performed using an Accu-scope fluorescence microscope.

Mean fluorescence intensity (MFI) was measured using Fiji-ImageJ software. The process involved opening the image from the “File” menu, then separating channels via the “Color” option in the “Image” menu. The green or red channel images were chosen for MFI measurement by selecting “Mean gray value” under “Set Measurements” in the “Analyze” menu. The polygon tool was applied to define the region of interest, and the MFI value was obtained by pressing “Ctrl + M” in the keyboard.

To see the myelin integrity and exposed axons, surface plot was drawn by using “interactive 3D surface plot version 3.24” module in ImageJ software.

Cell counting was conducted with CaptaVision + software (Accu-Scope Inc.). The image was opened and calibrated to 6 pixels/μm according to the raw image resolution. Using the “Measure” tool, the “Manual Class counting” function was selected, and cells were counted by clicking on each target cell.

### Immunoblot analysis:

Cells and tissue lysates were prepared with 5X Laemmli buffer, separated using 4–12% Tris–Glycine gels, and transferred to nitrocellulose membranes (P/N 926–31,090; Li-Cor Biosciences). Membranes were incubated overnight with primary antibodies, then for 2 h at room temperature with IRDye700/800-tagged secondary antibodies on an orbital shaker and imaged using the Odyssey Sa imager at 200 μm resolution.

### Live cell phagocytosis assay:

Approximately 80,000 adherent Mφ cells (counted in Countess^™^ automated counter) were added to 100 μL serum-free DMEM media mixed with 20 μL of pHrodo^™^ green BioParticles^™^ Conjugates (Cat# P35365; Thermo Fisher Scientific Inc) and then incubated at 37°C for 15 minutes. Afterward, the conjugate media was discarded, and the reaction was stopped by adding chilled methanol followed by storage at −20°C. Subsequently, cells were imaged under the FITC filter (480 nm) of an Accuris fluorescence microscope. For flow cytometry, cells were carefully scraped, centrifuged, and resuspended in 100 μL FACS buffer combined with 20 μL pHrodo^™^ green BioParticles^™^ Conjugates and other dye-tagged antibodies at a recommended dilution. After incubating at 37°C for 15 minutes, the cells were washed and subjected to flow cytometry analysis.

### siRNA transfection analysis:

Mouse splenic Mφs were transfected with 25 pmol sirt1 siRNA (Cat# 4390771; ThermoFisher Scientific) using lipofectamine 2000 per the manufacturer’s protocol in serum-free medium. After 4 hours, serum was added, and at 24 hours post-transfection, cells were treated with 0.5 mg/mL LPS. Sirt1 expression and activity were assayed 2 hours later.

### Sirtuin assay:

#### SIRT1 activity assay:

SIRT1 activity was measured in spleen lysates from transgenic and non-transgenic mice using the SIRT1 Activity Assay Kit (Fluorometric) (BPS Bioscience, Cat# 50081), following the manufacturer’s protocol. Briefly, spleens were harvested, rinsed in cold PBS, and homogenized in ice-cold lysis buffer (20 mM Tris-HCl, pH 7.5, 150 mM NaCl, 1 Mm Na 2 EDTA, 1mM EGTA, 1% Triton X-100, 2.5 mM Sodium pyrophosphate, 1mM β-glycerophosphate, 1mM Na3VO4, 1 μg/ml leupeptin with protease inhibitors) (Cell signaling technology Cat# 9803). Homogenates were centrifuged at 12,000 × g for 10 minutes at 4°C, and supernatants were collected. Protein concentration was quantified using the Bradford reagent (Thermo Fisher Scientific Cat# 1856209).

For the assay, 5 μL 100 μM substrate, 5 μL BSA (1 mg/ml), 5 μL 50 mM NAD+, 14.5 μl of SIRT assay buffer were added to each designated wells of a 96-well black microplate. Then, 20 μL of spleen lysate (containing 20 μg of total protein) from transgenic and non-transgenic mice was added. Each sample was run in triplicates. To this 50 μL of SIRT assay developer (2x) was added and the plate was incubated in room temperature for 15 minutes. Fluorescence was measured at Ex/Em = 385/485 nm at 60-second intervals for 30 minutes using Victor^™^ X3(PerkinElmer) a microplate reader.

#### SIRT2 activity assay:

SIRT2 activity was measured in spleen lysates from transgenic and non-transgenic mice using the SIRT2 Activity Assay Kit (Fluorometric) (Abcam, Cat# ab156066), following the manufacturer’s protocol. Briefly, spleens were harvested, rinsed in cold PBS, and homogenized in ice-cold lysis buffer as mentioned in SIRT1 assay procedure. Tissue lysates were centrifuged at 12,000 × g for 10 minutes at 4°C, and supernatants were collected. Protein concentration was quantified using the Bradford reagent (Thermo Fisher Scientific Cat# 1856209).

For the assay, 25 μL of double distilled water, 5 μL of SIRT2 assay buffer, 5 μL of fluoro- substrate peptide, 5 μL of NAD+ solution and 5 μL of developer solution were added to each designated wells of a 96-well black microplate. Then, 5 μL of spleen lysate (containing 20 μg of total protein) from transgenic and non-transgenic mice was added. Each sample was run in triplicates. Fluorescence was measured at Ex/Em = 485/535 nm at 120-second intervals for 30 minutes using Victor^™^ X3(PerkinElmer) a microplate reader.

### Seahorse mitochondrial OCR and Glycolytic ECAR analyses.

Measurement of mitochondrial oxygen consumption and glycolysis in Mφ cells were performed by using a Seahorse XF96 extracellular flux analyzer as described elsewhere (). Briefly, Mφs were isolated from spleens of 10–12 weeks ‘old male NTg and age-/gender- matched Tg^+/−ATG13^ mice (n=3/group), suspended in Agilent-recommended seahorse RPMI media (part # 103576–100), seeded in a Seahorse XF96 96-well microplate (part #102959–10096) at a concentration of 200,000 cells/well. To proper attachment in the bottom, cells were spun down, and the media was replaced with fresh RPMI media. Oxygen measurements were started, and at the specified time points, the solutions different mitochondrial and glycolytic inhibitors were added. The data was displayed at Seahorse Wave software and then exported to GraphPad Prism using a dropdown menu under export tab.

For post-treadmill seahorse OCR and ECAR study, age-matched (n=3) male were subjected to treadmill exercise for 14 rpm for 15 minutes. After 4 hrs, splenic Mφs were isolated, counted, plated with OCR/ECAR media and immediately measured for OCR/ECAR assay. The result was compared with splenic Mφs of age/gender/genotype-matched mice (n=3) without treadmill exercise.

### Treadmilling, Open-field and EMG analyses:

*Treadmilling*, Open field and EMG recording were performed as discussed before [13].

A Columbus XR4 mouse treadmill (S/n APM933AT-I) was used to assess muscle fatigue. Each mouse was subjected on the motorized belt at 5 rpm for 1 minute, then at 15 rpm for 14 minutes. Cardboard blocked the track’s end to prevent escape.

Following treadmill exercise, each mouse underwent gross movement assessment in an open-field acrylic arena (Stoelting Co; Cat # 60,100). The arena measures 40 cm on each side and features transparent walls as well as a detachable fiber base for cleaning. Movement was recorded using a Stoelting digital USB camera (Cat#10–000–332) mounted on the ceiling via a suspension bar. The camera was connected to ANY-maze video tracking software with a cable. For each trial, horizontal activity, total distance moved, movement time, resting time, and tracking plot were measured. Each recording began after a 2-minute acclimatization period.

EMG recordings were obtained from both non-transgenic (NTg) and Tg+/−ATG13 mice using AD Instruments PowerLab, amplified with Bio Amp PowerLab, and sampled at 2 kHz. Results were displayed in Chart software at 10–50 μV resolution, with reference values ranging from −5 to 5 mV.

### Statistical analysis.

Sample size n =8 is calculated with the help of a statistical calculator based on confidence interval 0.95, margin of error 5%, and the population proportion = 0.5. The significance of mean between groups was analyzed in GraphPad Prism 10 software by using unpaired t-test (parametric; 2 groups)), Mann-Whitney U test (non-parametric; 2 groups), a one-way ANOVA (parametric and one effector; more than 2 groups) or two-way ANOVA (parametric and one effector; more than 2 groups) combined with Tukey’s HSD multiple comparison tool. The decision of conducting either parametric or non-parametric tests was made after assessing the normality distribution Q-Q plot followed by D’Agostino & Pearson test(*p<0.05). Data were presented as mean ± SEM and the significance was deemed acceptable at p<0.05.

## Results

### Depletion of atg13 gene in Tg-ATG13 mice inhibits macroautophagy in splenic Mφ cells.

**Figure 1:**
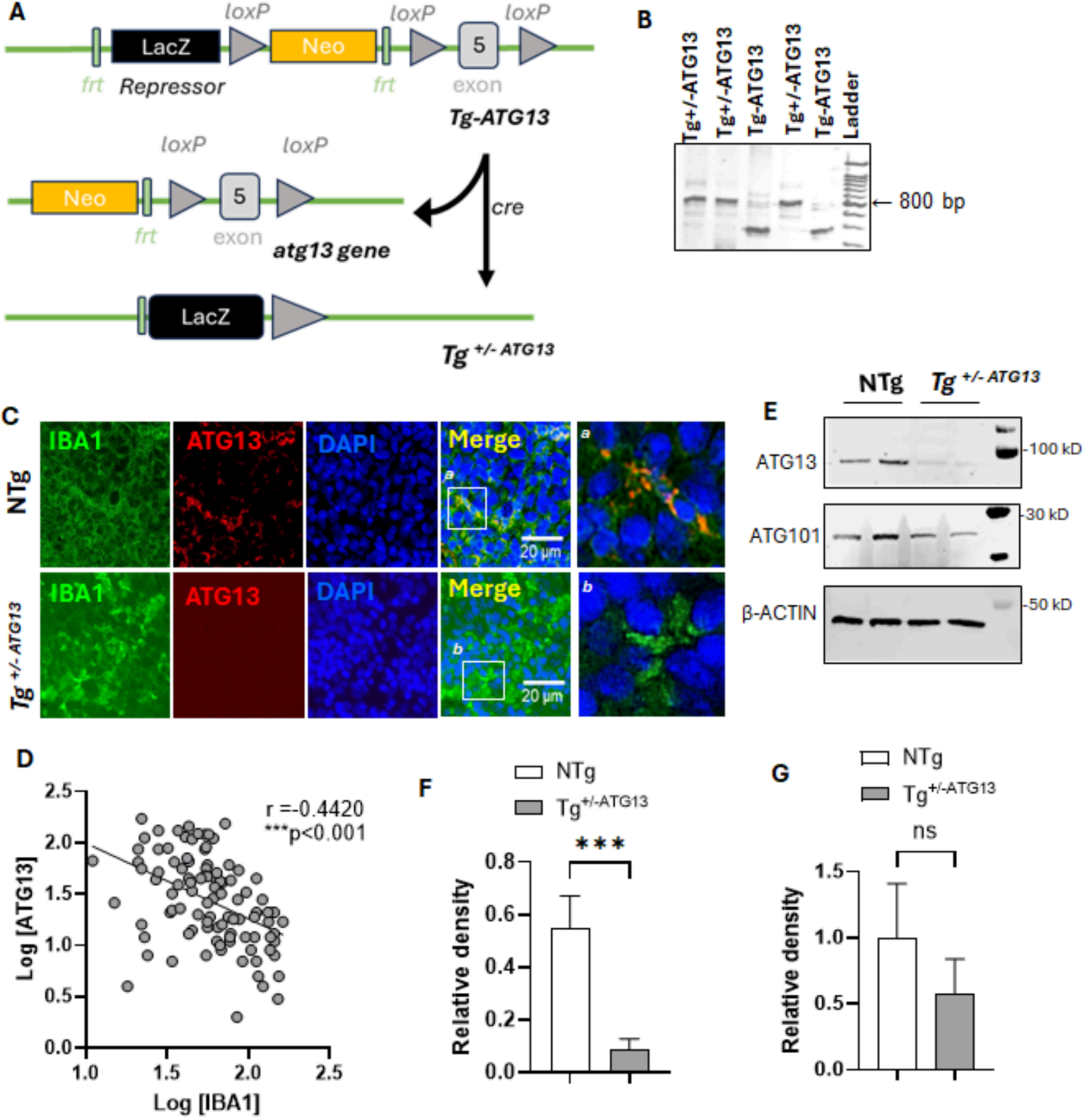
Generation of Tg^+/−ATG13^ mice and evaluation of ATG13 expression in spleen. (A) A schematic strategy for the generation of Tg+/−ATG13 mice. (B) The genotype data with different primer sets (as mentioned in Method section) for identifying LacZ repressor Tg-ATG13 (~500 bp=450 bp) and Tg^+/−ATG13^ (~800 bp=797 bp) mice. Non-transgenic (NTg) mice did not show any band neither at ~500 nor ~800 bp. (C) Splenic tissue of 10 weeks ‘old male NTg and Tg+/−ATG13 (n=5/group) were paraffin-embedded, cut at 5 μm thickness, and double immunostained with ATG13 and activated Mφ marker IBA1. (D) The parametric Pearson’s correlation was performed to monitor the relationship between IBA1 and ATG13 expressions in 100 dual-stained cells. Liner regression plotted in GraphPad Prism 10 indicates that the slope (Pearson coefficient=r=−0.4420) is significantly non-zero with F1,98 =23.80; **p<0.001. (E) Immunoblot analyses of ATG13, ATG101, and loading control β-actin were shown. (F) The relative densities of (F) ATG13 and (G) ATG101 were calculated as a ratio of target band intensity with respective actin band density and plotted as histograms. Mann Whitney U test was performed to test the significance of mean between groups that results in ***p<0.001 vs. ATG13 of NTg. NS=nonsignificant. Results are confirmed after three independent experiments from n =5 animals/group.

Previously, our research demonstrated that ATG13 repressor mice (Tg-ATG13) with lacZ repressor-directed suppression of *atg13*, an essential autophagy gene, strongly impaired autophagy and induced inflammatory demyelination in muscle-serving nerve fibers. The underlying molecular mechanism was unclear. To pinpoint the molecular role of atg13 gene, we developed a cre-loxP conditional knock-out ATG13 mice (Tg^+/−ATG13^) (Fig. 1A) with a hemizygous deletion of critical exon 5 of *atg13* gene (Fig. 1B).These female Tg^+/−ATG13^mice displayed significantly enlarged spleen indicting that *atg13* gene-depleted splenic cells may be associated with altered immune metabolism. To understand the role of ATG13 in metabolic deficits in immune cells especially in macrophages (Mφ), we first performed a dual immunostaining of ATG13 along with inflammatory Mφ marker IBA1 (Fig. 1C) in spleen. The dual IF analysis together with Pearson correlation statistics (Fig. 1D) in splenic sections followed by an IB analysis in purified splenic Mφ cells (Fig. 1E & 1F) showed that the hemizygous depletion of atg13 gene in Tg^+/−ATG13^significantly reduced ATG13 expression in IBA1-ir inflammatory Mφ cells, but not non-transgenic (NTg) mice, indicating that the depletion of the *atg13* gene may promote inflammation in Mφ cells. Interestingly, the expression of another autophagy protein ATG101, which forms a complex with ATG13 to initiate autophagosome formation, did not change (Fig. 1E & 1G) significantly.

**Figure 2:**
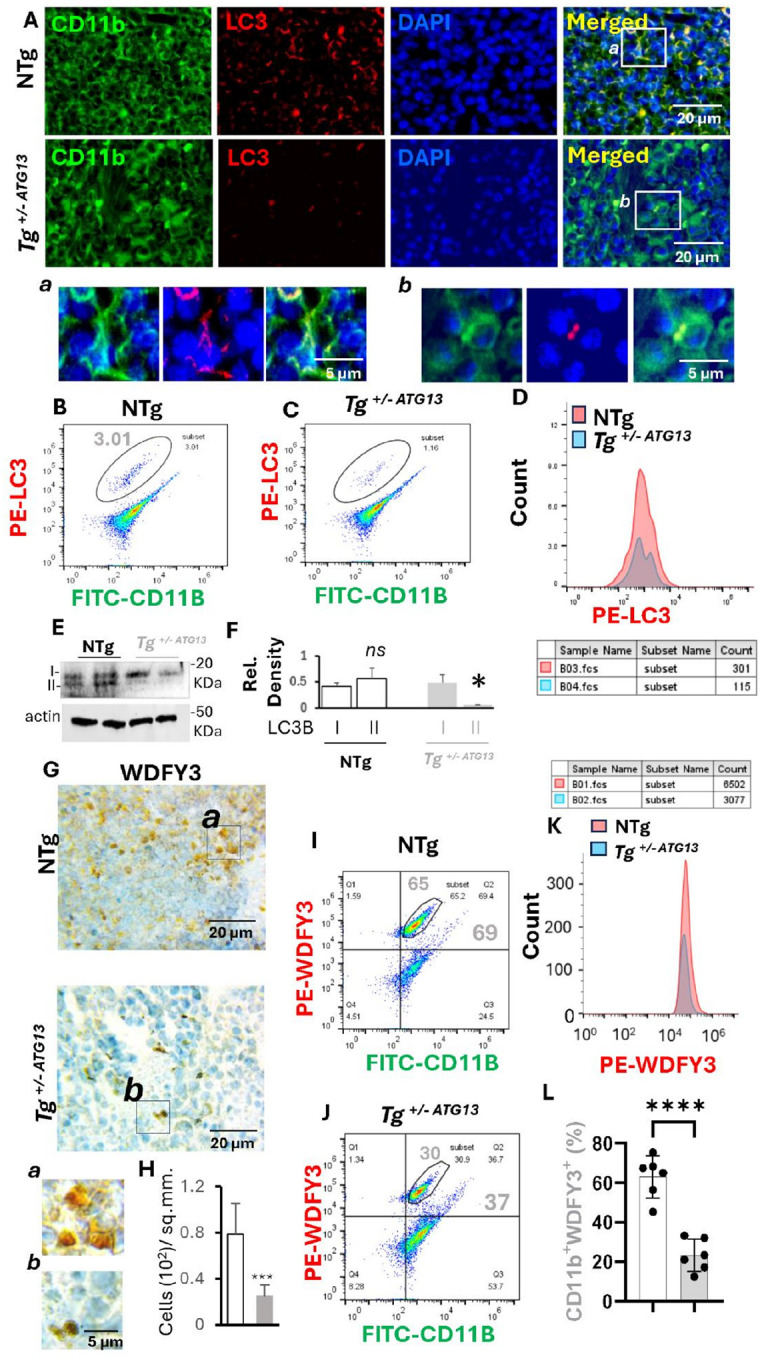
Impairment of autophagy in splenic Mφ of Tg^+/−ATG13^ mice. (A) Dual IF analysis of LC3 (Rabbit anti-LC3; Cat#; ProteinTech; dilution 1:100) and CD11b (Mouse anti-CD11b; Cat#; Invitrogen; dilution 1:100) in 5 μm thick paraffin-embedded sections of 10–12 weeks old non-transgenic and hemizygous atg13 knockout mice (n=6/group). (*Inset*) The magnified view dual IF images of (a) NTg and (b)Tg^+/−ATG13^. Dual flow-cytometry of PE-labeled LC3 and FITC-labeled CD11b in purified Mφs isolated from n=6 10–12 weeks ‘old (B) NTg and (C) Tg^+/− ATG13^ mice (male+female). The total gated events are 20,000/group. The cells under enclosed area represent a distinct population of CD11b-ir cells, which is also LC3-positive. (D) Histogram analyses to quantify the CD11b^+^LC3^+^ cells in NTg and Tg^+/−ATG13^ Mφs. (E) Immunoblot analyses of LC3II demonstrates two distinct bands of autophagy-active LC3BII (lower) and autophagy-inactive LC3BI (upper) bands. Beta-actin immunoblot was run as loading control. (F) The relative densitometric analysis was plotted after normalizing with respective β-actin bands, White and grey bars represent LC3B band densities of NTg and Tg^+/−ATG13^ mice respectively.(Unpaired t-test was run to test the significance of mean between groups that displayed *p<0.05 vs. LC3BI of Tg^+/−ATG13^. NS = not significant. (G) DAB immunostaining of WDFY3 (Rabbit; cat#, Invitrogen, dilution) in splenic sections (n=5/group) counterstained with hematoxylin. (inset) Enclosed zones were magnified (a =NTg, b= Tg). (H) Counting of WDFY3+ cells was performed in 5 different images from n=5 mice/group following the quantification per sq.mm. of sections (Unpaired t test shows ***p<0.005 = 0.002844221). (I & J) Representative Flowcytometry analyses of WDFY3 (PE-tagged) and CD11b (FITC-tagged) (Gated events 10000). (K) Histogram counting analysis of encircled population with numbers of gated cells. (L) Scatter bar graph shows the counts of CD11b+WDFY3+ cells in five images per group (unpaired t test shows ****p<0.0005). Results are mean ± SD of three experiments.

To understand the molecular role of atg13 in regulating the cellular metabolism of autophagy in Mφ cells, following assays were performed. *First*, a dual IF analysis (Fig. 2A) of pan Mφ marker CD11b and general autophagy marker LC3 indicated a strong reduction of autophagosomes in Mφ cells of Tg^+/−ATG13^spleen compared to same of NTg mice. *Second*, dual flow-cytometry analyses (Fig. 2B–D) of CD11b and LC3 also displayed a strong and significant reduction () of LC3-ir population in the splenic Mφ cells of Tg^+/−ATG13^mice. *Third*, the functional significance of LC3 upregulation in autophagy was further evaluated by IB analysis (Fig. 2E) followed by quantifying the level of LC3IIb over LC3IIa (Fig. 2F) in splenic Mφ cells, which indicated a significant reduction of LC3IIb in Tg^+/−ATG13^Mφ cells. *Fourth*, to evaluate the role of ATG13 in overall autophagy flux, a DAB immunostaining (Fig. 2G) of macroautophagy marker WDFY3 followed by quantification analysis (Fig. 2H) were performed, which revealed a significant loss of autophagy in Tg^+/−ATG13^Mφ cells. *Fifth*, flow-cytometry study (Fig. 2I–2J) coupled with quantification analysis (Fig. 2K–2L) further indicated that the WDFY3-ir population was significantly reduced in purified Tg^+/−ATG13^Mφ cells. Finally, the reduction of WDFY3 in purified splenic Mφ cells was confirmed by IB analysis reiterating that the genetic suppression of *atg13* gene indeed inhibited autophagy in Mφ cells.

### The effect of atg13 gene suppression on the polarization of M1Mφ cells.

**Figure 3:**
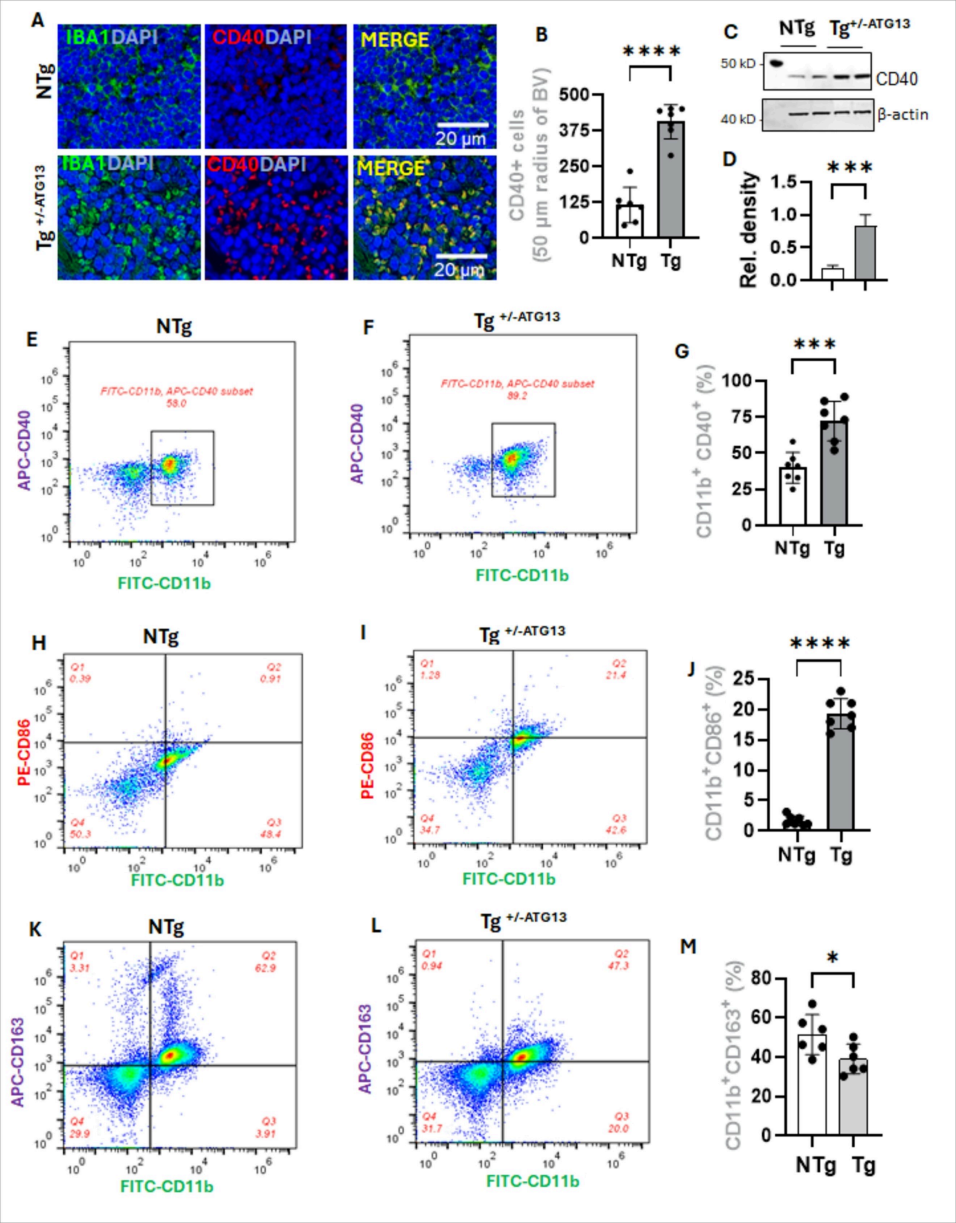
polarization of M1Mφ cells in spleen of Tg^+/−ATG13^ mice. (A) Dual IF analysis of CD40 (Rabbit anti-CD40; Cat#; ProteinTech; dilution 1:100) and IBA1 (Mouse anti-IBA1; Cat#; Invitrogen; dilution 1:100) in 5 μm thick paraffin-embedded spleen sections of 10–12 weeks old male NTg and Tg^+/−ATG13^ mice (n=6/group). (B) Quantification of CD40-ir cells were performed in 50 μm radius of blood vessel in red pulp zone. An unpaired t-test was performed to verify the significance of mean between group that results in ****p<0.0005. Results were confirmed after counting 7 independent images per group. (C) IB followed by (D) β actin-normalized densitometric analyses displayed ***p<0.005 (unpaired t-test) between groups. Dual flow-cytometry of APC-labeled CD40 and FITC-labeled CD11b in purified Mφs isolated from n=6 (male+female) 10–12 weeks ‘old (E) NTg and (F) Tg^+/− ATG13^ mice. The total gated events are 20,000/group. (H &I) Similar dual flowcytometry analysis between CD86 (PE-tagged; dil 1:100) and CD11b (FITC-tagged; dil 1:100) followed by (J) quantification (n=7 analysis/group) were performed (****p<0.0005; Unpaired t test). (K &L) Dual flowcytometry of CD163 (APC-tagged; dil 1:100) and CD11b (FITC-tagged; dil 1:100) followed by (M) quantification (n=7 analysis; ****p<0.0005 by unpaired t test) analysis were performed in purified Mφ cells. Results are mean ± SD of three experiments.

How does the suppression of atg13 gene affect Mφ cell properties? During inflammation, Mφ cells acquire inflammatory M1 properties, which can be evaluated by analyzing expressions of a wide range of surface proteins. One such traditional M1 surface protein CD40 was immunolabeled (Fig. 3A) along with inflammatory marker IBA1 in splenic tissue of Tg^+/−ATG13^and NTg mice. Interestingly, both IBA1 and CD40-ir cells (Fig. 3B) were observed to be significantly upregulated in ^Tg+/−ATG13^spleen. The result was further confirmed by IB analysis (Fig. 3C) in purified Mφ cells followed by the relative densitometric quantification study (Fig. 3D) and flowcytometry (Fig. 3E–F) followed by quantification (Fig. 3G) analyses. The role of *atg13* depletion in acquisition of M1 phenotype was further confirmed by dual flowcytometry analysis of another M1 marker CD86 with CD11b (Fig. 3H–I) followed by quantification analysis (Fig. 3J). Polarization of Mφ to M1 phenotype has been often shown to be associated with the deviation from anti-inflammatory M2 phenotype. To test, we performed dual flowcytometry analysis of M2 marker CD163 and pan Mφ marker CD11b (Fig. 3K–L) in purified splenic Mφ followed by the quantification analysis (Fig. 3M). The results clearly indicate that there is a significant reduction of M2 surface properties in Mφ cells of ^Tg+/−ATG13^spleen. Collectively, our results indicate that the suppression of atg13 gene strongly induced the polarization of M1 phenotype of Mφ cells.

**Figure 4:**
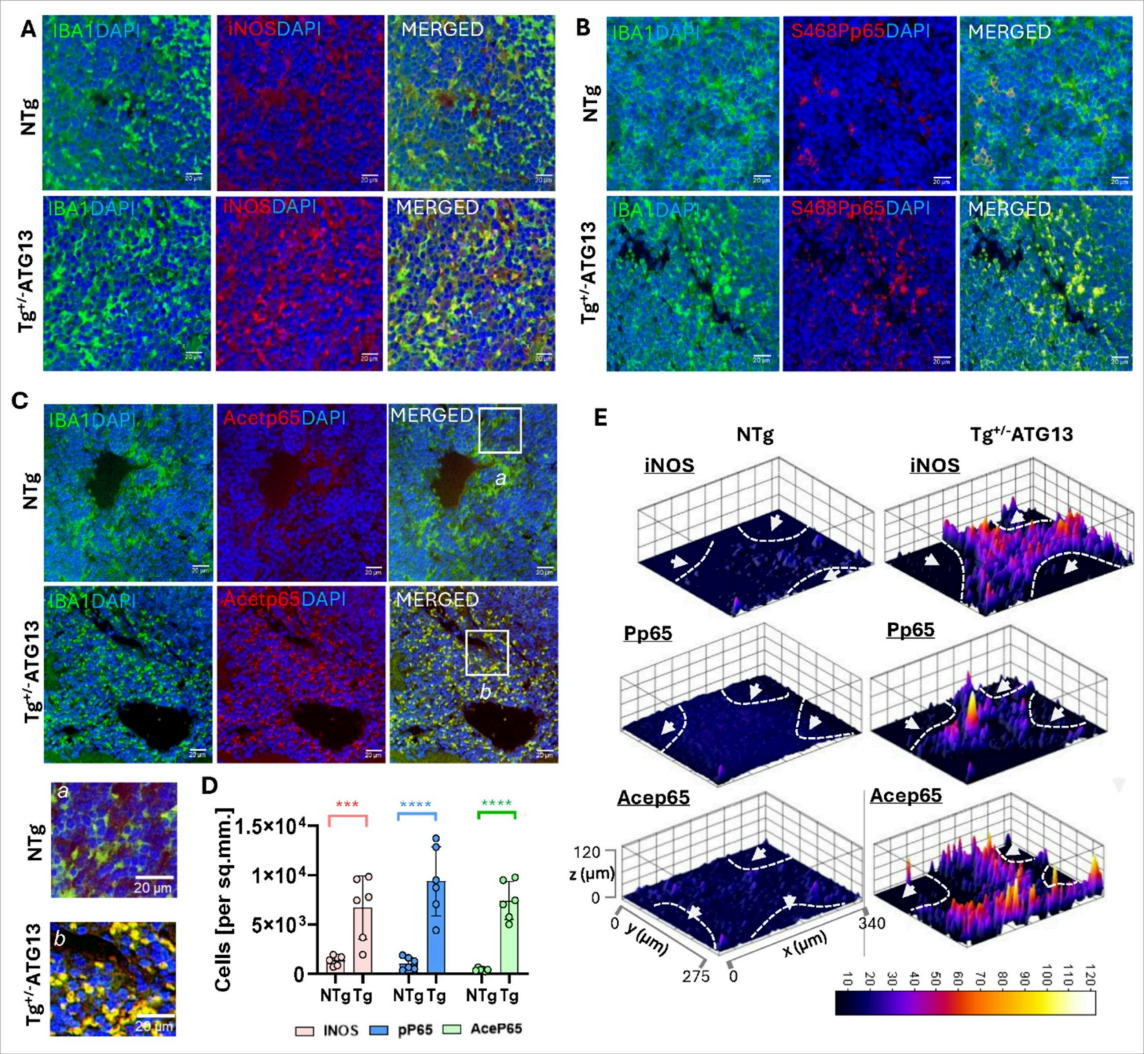
Functional Characterization of M1Mφ cells in spleen of Tg^+/−ATG13^ mice. Dual IF analysis of IBA1 (Mouse anti-IBA1; Cat#; Invitrogen; dilution 1:100) with M1Mφ functional markers including (A) iNOS (Rabbit anti-CD40; Cat#; ProteinTech; dilution 1:100), (B) Serine468phospho (S468P) NFκB subunit p65, and (C) Acetylated p65 in paraffin-embedded spleen sections of 10–12 weeks old NTg and Tg^+/−ATG13^ mice (n=6/group). (*inset*) The enclosed regions of acetylated p65 and IBA1–ir cells were magnified for (a)NTg and (b) Tg spleen. (D) Quantification analyses of iNOS (pink bars), S468Pp65 (blue bars), and acetylated p65 (green bars) in n=6 independent images. Unpaired t tests to test the significance of mean result in ***p<0.005 and ****p<0.0005 versus NTg. (E) 3D Surface plots (ImageJ software) were drawn to visualize fluorescence intensities and the distribution of iNOS, S468Pp65, and acep65-ir cells in the red pulp regions. Red pulp regions were separated from white pulp regions (arrow-head) by dotted white line. Distinctively less distribution of immunoreactive cells were observed in white pulp regions. Results are mean ± SD of three experiments.

Next, we are interested to study if atg13 depletion stimulated the function of M1Mφ cells. Functionally, M1Mφ cells are associated with upregulations of inflammatory mediators such as inducible nitric oxide synthase (iNOS)[22] as well as Serine 468 phosphorylated (S468P) and acetylated p65 subunits of nuclear factor kappa B (NF-κB)[23–25]. A series of dual IF studies indicated that IBA1-ir cells of ^Tg+/−ATG13^mice strongly expressed iNOS (Fig. 4A), S468P p65 (Fig. 4B), and acetylated p65 (Fig. 4C). The result was further corroborated by quantification analysis (Fig. 4D). To map the distributions of these inflammatory cells and expression patterns of these mediators we next performed surface plot analyses (Fig. 4E). Accordingly, we observed that background-adjusted mean fluorescence intensities of iNOS, S468Pp65, and acetylated-p65 were significantly higher in Tg^+/−ATG13^spleen compared to NTg. Additionally, these Mφ cells were found to be specifically distributed in the red pulp, but not in the white pulp (as indicated by arrowhead within dotted line suggesting marginal zone) regions of spleen. This observation suggests that the depletion of *atg13* gene led to the polarization and localization of M1Mφ cells near the vasculature of splenic tissue, potentially contributing to a systemic inflammatory response.

**Figure 5:**
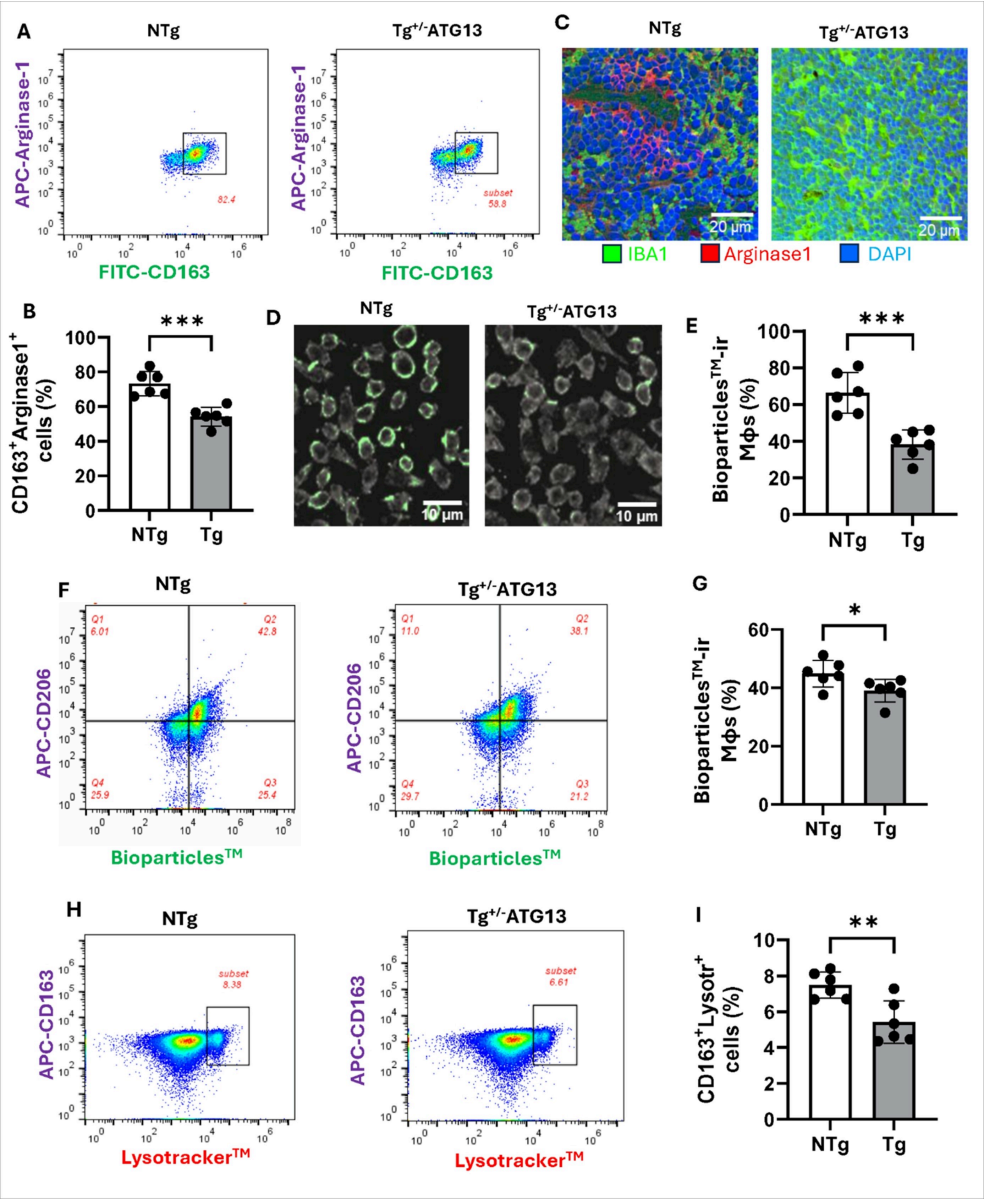
Functional Characterization of M2Mφ phenotype in splenic Mφ cells of Tg^+/−ATG13^ mice. (A) Dual IF analysis of M2Mφ functional marker arginase-1 (APC-tagged) with M2Mφ surface marker CD163(FITC-tagged). The enclosed subsets identify CD163+ arginase1+ cells in purified splenic Mφ cells. Total gated events =10000. (B) Scatter histogram displays counting of CD163+Arginase1+ cells in n=6 analyses/group. Unpaired t test indicates the significance of mean between groups with ***p<0.005. (D) Phagocytosis assay of live Mφ cells by immunocytochemistry of pHrodo^™^ green conjugated BioParticles^™^ (Cat#P35381; ThermoFisher; 10^6^ cells in 10,000 Mφ cells) uptake. (E) Quantification of phagocytic cells in n=6 images/group followed by unpaired t test to verify the significance resulting in ***p<0.005. (F) Dual flowcytometry of CD206 (APC-tagged) and Bioparticles^™^ (pHrodogreen-tagged) followed by (G) quantification of Bioparticles^™^ (%)-conjugated CD206^+ve^ cells (n=6 analyses). Unpaired t test to verify the significance of mean indicates *p<0.05. Total gated events = 20000. (H) Dual flowcytometry of CD163 (APC-tagged) and Lysotracker^™^ (Red-DND99-tagged detected in PE filter; ThermoFisher; Cat# pHrodogreen-tagged) followed by (I) quantification of Lysotracker^^™^^™^^ (%)-conjugated CD163^+ve^ cells (n=6 analyses). Unpaired t test to verify the significance of mean indicates *p<0.05. Total gate =50000. Results are mean ± SD of three experiments.

Does the suppression of atg13 inhibits the function of M2Mφ cells? Given that our results indicate atg13 suppression may impede the polarization of M2Mφ cells, we proceeded to investigate whether the associated metabolic functions of M2Mφ cells are similarly inhibited. First, the neutralization of nitric oxide by arginase, a key metabolic event of M2Mφ cells, is evaluated by measuring the expression of arginase via flowcytometry. Accordingly, a dual flowcytometry (Fig. 5A) of arginase with M2Mφ marker CD163 indicated a strong suppression of CD163^+^Arginase^+^ cells (Fig. 5B) in purified splenic Mφ cells of Tg^+/−ATG13^mice compared to NTg mice. Next, dual IF analyses of arginase and IBA1 (Fig. 5C) also demonstrated that there is a strong reduction of arginase in IBA1-ir Mφ cells of Tg^+/−ATG13^spleen. Another functional property of M2Mφ cells is phagocytosis that can be evaluated by zymosan green assay, in which live Mφ cells were fed with green fluorescence-tagged zymosan particles. Fluorescence-based zymosan engulfment assay (Fig. 5D) followed by counting (Fig. 5E) and then a dual flowcytometry assay of zymosan- green along with M2Mφ marker CD206 (Fig. 5F–G) clearly demonstrated that atg13 depletion strongly attenuated the phagocytic property in isolated splenic Mφ cells of Tg^+/−ATG13^mice. Lysosomal function, as an indicator of autophagy, is significantly suppressed in Tg^+/−ATG13^splenic Mφ cells based on a dual flow cytometry study of lysotracker and CD163 (Fig. 5H) followed by a quantification analysis (Fig. 5I). Collectively, these results suggest that *atg13* gene suppression not only upregulated the expressions of M1 surface markers but also induced the inflammatory properties of splenic Mφ cells.

### Genetic ablation of atg13 impairs redox metabolism in mitochondria resulting in the activation of NFκB via nitrotyrosination of SIRT1 enzyme.

**Figure 6:**
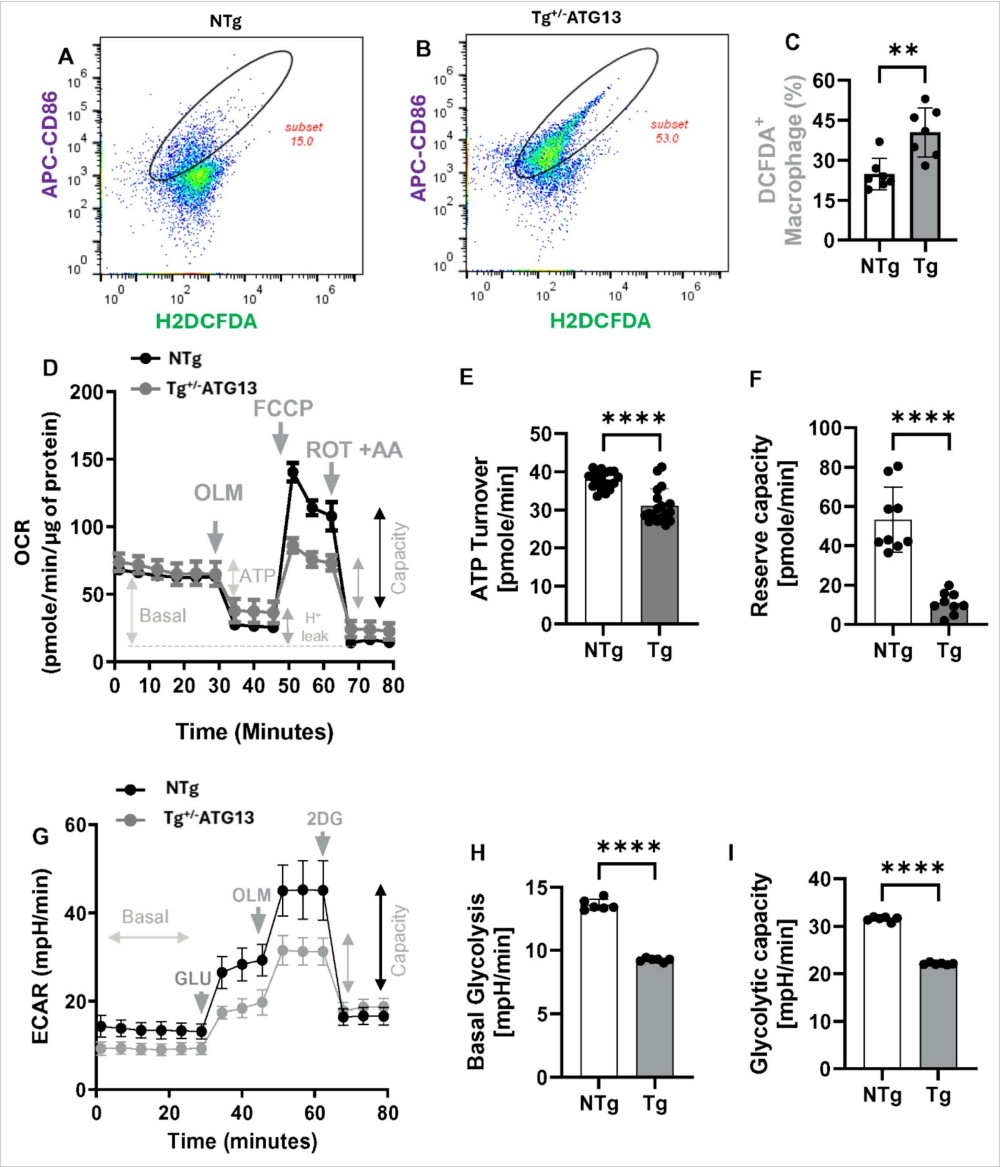
Production of ROS and evaluation of metabolic events responsible for ROS production in Mφ cells of Tg^+/−ATG13^ mice. (A) Dual IF analysis of M1Mφ surface marker CD86 (APC-tagged) with ROS sensor H2DCFDA (FITC-filter). The enclosed ellipsoids are subsets of ROS-producing population in purified splenic Mφ cells. Total gated events =10000. (B) Scatter histogram displays counting of DCFDA+ cells in n=6 analyses/group. Unpaired t test indicates the significance of mean between groups with **p<0.01. (D) Seahorse metabolic assay of mitochondrial oxidative phosphorylation (OXPHOS) was evaluated in XF96 equipment (Agilent) by measuring oxygen consumption rate (OCR) followed by the visualization and analysis in Agilent Wave^™^ software. Approximately 200,000 cells were treated with OLM = Oligomycin (2 μg/mL); FCCP = Fluoro-carbonyl cyanide phenylhydrazone (2 μM), ROT=rotenone, and AA=antimycin A. (E) ATP utilization and (F) reserve capacity indicative of the capacity of performing OXPHOS under stress was evaluated by scatter histogram analyses. ****p<0.0005 versus NTg based on unpaired t test. (G) Glycolysis was evaluated by measuring extracellular acidification rate (ECAR) in Seahorse XF96 followed by displaying in Wave_^™^_ software. GLU = Glucose, 2DG = 2 Deoxy-glucose. Scatter histogram analyses of (H) basal glycolysis and (I) glycolytic capacity. Unpaired t test revealed ****p<0.0005 versus NTg. Results are mean ± SD of three experiments.

Next, we were interested to study the molecular mechanism of how the ablation of atg13 gene induced the polarization of M1Mφ cells. The atg13 ablation-mediated loss of autophagy may directly compromise mitochondrial function of energy metabolism resulting in the production of ROS. In fact, splenic M1Mφ cells of Tg^+/−ATG13^mice displayed upscaled ROS production as indicated by dual flowcytometry assay of ROS-sensor DCFDA with M1 marker CD86 (Fig. 6A–C) suggesting the potential deficit of mitochondrial energy metabolism in Tg^+/−ATG13^Mφ cells. To explore the direct role of *atg13* gene ablation in the mitochondrial deficit of energy metabolism, next we performed a seahorse oxygen consumption assay (Fig. 6D) with different mitochondrial inhibitors as described elsewhere[26]. Interestingly, mitochondrial oxygen consumption rate (OCR) measurement assay in purified splenic Mφ cells displayed no difference in basal respiration (before oligomycin or OLM treatment). However, there was a significant drop in ATP turnover (Fig. 6E) in Tg^+/−ATG13^Mφ cells indicative of less bioavailability of ATP compared to splenic Mφ cells of NTg mice. Additionally, Tg^+/−ATG13^Mφ cells showed a significant reduction in reserve capacity (Fig. 6F), indicating higher susceptibility to impaired mitochondrial energy production during stress. Next, we performed glycolysis assay (Fig. 6G) in Mφ cells by measuring extracellular acidification rate (ECAR) using different glycolytic inhibitors as described elsewhere (27911401). Both basal glycolysis (Fig. 6H) and glycolytic reserve (Fig. 6I) suggesting that the genetic ablation of the atg13 gene in Tg^+/−ATG13^mice significantly reduces glycolysis in Mφ cells both under basal conditions and stress. Collectively, our mitochondrial and glycolytic metabolic assay demonstrated that *atg13* gene ablation significantly impaired the basal energy metabolism of glucose and production of ATP. Moreover, these deficits may be exacerbated during stress.

**Figure 7:**
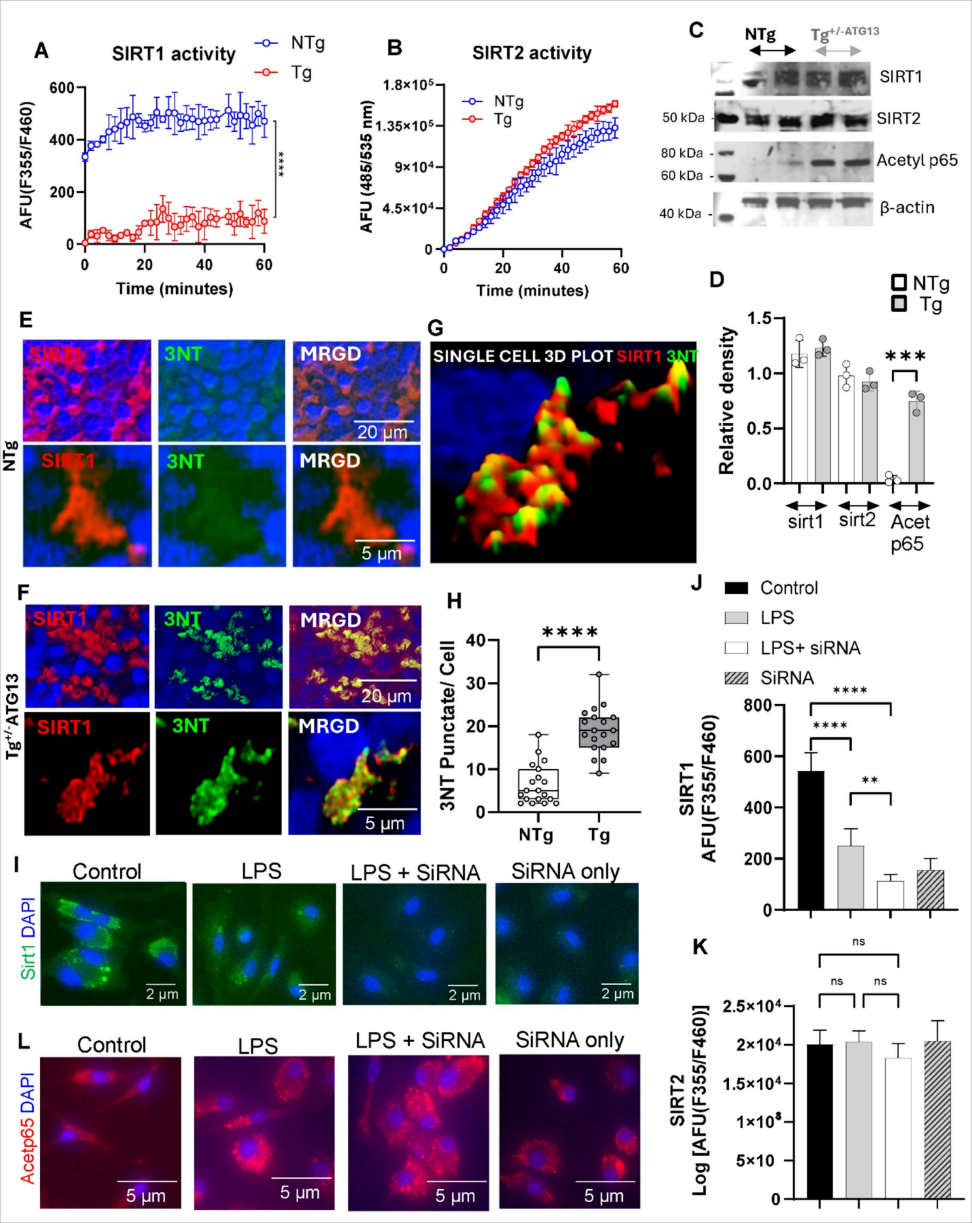
The ROS–mediated inhibition of Sirtuin-1, but not Sirtuin-2, may be responsible for inflammation. (A) A fluorescence-based (EX: Em = 355/460 nm) enzyme assay of sirtuin-1 (SIRT1) in splenic Mφ cells. The assay was performed as instructed by the manufacturer (BPS Biosciences) using a cell lysate containing 1 μg of total protein. (B) Fluorescence-based sirtuin-2 assay per the manufacturer’s protocol (Abcam) with 1 μg of protein derived from the cell lysate of Mφ cells. (C) IB analyses followed by (D) densitometric analyses of sirt-1,2, acetylated p65, and β-actin in splenic Mφ cells of NTg and Tg^+/−ATG13^ mice (n=3 pr group). Dual IF analyses of SIRT-1 (red) and 3-nitrotyrosine (3NT; green) in the spleen of 10–12 weeks ‘old (E) NTg and (F) Tg^+/−ATG13^ mice (n=6/group). (G) 3D visualization of a single cell (ImageJ) displays the juxtaposition of SIRT1 and 3NT. (H) Quantification followed by scatter boxplot analysis, analyzing the total number of 3NT-ir puncta per cell in 19 cells /group. Unpaired t test indicates ****p<0.0005 versus control. (I) Tg splenic macrophages were transfected with siRNA against Sirt1 (250 pmol; Cat # AM16708; ThermoFisher Scientific, MA), followed by treatment with 0.5 mg/mL LPS for 2 hrs, and then immunostained with sirt1 antibody to check the efficiency of siRNA. Enzyme activity assay of (J) SIRT1 and (K) SIRT2 after 30 minutes of incubation with siRNA- transfected and non-transfected cell lysates (1 μg protein).(L) IF analysis of acetylated p65 in Tg splenic Mφ cells transfected with sirt1 siRNA followed by the treatment with 0.5 mg/mL LPS. Results are the mean ± SEM of three different experiments.

Next, we were interested to study how the molecular deficit of energy metabolism contributed to the inflammatory properties of splenic Mφ cells of Tg^+/−ATG13^mice. Our results indicated that acetylation of p65 was markedly increased due to the ablation of atg13. Recent literatures also suggest that a class of cellular deacetylases known as sirtuins (SIRT) especially isoform1 (SIRT1)[27] and 2 (SIRT2)[28] regulate deacetylation of p65[29] and therefore known for their roles in the suppression of inflammation. Interestingly, we observed that activity of SIRT1(Fig. 7A), but not SIRT2 (Fig. 7B), markedly decreased in Tg^+/−ATG13^splenic Mφ cells. However, the cellular expression of Both SIRT1 and SIRT2 did not alter due to ATG13 depletion as confirmed by IB (Fig. 7C) followed by relative densitometric analyses (Fig. 7D). The reduction in SIRT1 enzymatic activity may be attributed to post-translational modifications, such as nitrosylation, potentially arising from elevated oxidative stress within the mitochondria. To explore that, we performed dual IF analyses of SIRT1 and 3-nitrotyrosine (Fig. 7E–F) followed by a 3D colocalization mapping (Fig. 7G). Interestingly, a notable colocalization of SIRT1 and 3-nitrotyrosine in Tg^+/−ATG13^spleen (Fig. 7H) suggests that the loss of function of SIRT1 may be attributed to the nitrosylation of tyrosine residue as a response to oxidative stress following depletion of the atg13 gene. To further confirm, we performed siRNA mediated silencing of *sirt1* gene in splenic Mφs by a commercially available (ThermoFisher) siRNA. Accordingly, SIRT1 expression (green puncta, Fig. 7I) and activity (Fig. 7J), but not SIRT2 (Fig. 7K), were markedly reduced in sirt1 siRNA-transfected, LPS-activated Tg^+/−^ATG13 splenic Mφs, indicating effective sirt1 gene silencing by the siRNA strategy. Interestingly, we observed that the silencing if sirt1 gene strongly upregulated acetylated NFκB in LPS-activated Tg^+/−^ATG13 Mφs (Fig. 7L) suggesting that the absence of ATG13 critically downregulated the enzymic activity of SIRT1 to stimulate the activation of NFκB.

**Figure 8:**
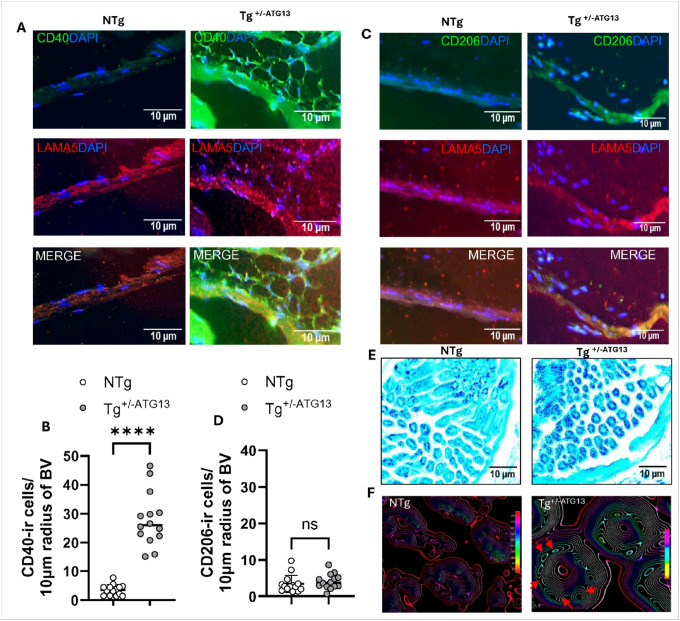
The effect of atg13 ablation on the perivascular infiltration of M1Mφ cells and compromised myelin integrity. (A) Dual IF analysis of M1Mφ marker CD40 (green; dil 1:100) and blood vessel endothelium marker laminin alpha-5 (LAMA5; red; 1:100) (B) Scatter histogram analyses of CD40-ir cells at a 10 μm radius from the outer wall of the blood vessel. Unpaired t-test represents the significance of the mean between two groups with ****p<0.0005 by counting n =13 vessels/group. (C) Dual IF analysis of M2Mφ marker CD206 (green) and LAMA5 (red). (D) Scatter histogram analyses of CD206-ir cells at a 10 μm radius from the outer wall of the blood vessel. NS = not significant by counting n =13 vessels/group. (E) LFB staining of myelin (lighter blue) in the muscle-serving nerve bundles counterstained with cresyl violet (deep blue) in the biceps femoris muscle of NTg and Tg ^+/−ATG13^ mice (n =5/group). (F) 3D surface plot (ImageJ) identified axonal fibers (cyan circles labeled with red arrowhead) with less myelin integrity. Results are mean ± SEM of three different experiments.

Previously, our study[13] delineated that the suppression of atg13 gene induced muscle fatigue in mouse. To explore the molecular mechanism, we investigated if there is an augmented infiltration of these polarized M1Mφ cells in the muscle vasculature of Tg^+/−^ATG13 mice. Interestingly, a dual IF analysis of blood vessel endothelium marker laminin alpha5 (LAMA5) and M1Mφ marker CD40 (Fig. 8A) following a quantitative measurement with 10 μm periphery of blood vessel tract (Fig. 8B) revealed that there was an upregulated infiltration of CD40-ir Mφ cells. However, while immunostained (Fig. 8C) followed by quantified (Fig. 8D) with M2Mφ marker CD206, these cells displayed no immunoreactivity suggesting that the infiltrated Mφs are primarily of M1, not M2 phenotype. Furthermore, an LFB staining (Fig. 8E) to evaluate the myelin integrity in the nerve-bundle of skeletal muscle (biceps femoris) demonstrated that the genetic depletion of atg13 gene significantly reduced the myelin thickness (cyan) and increased the numbers of exposed axonal fibers (deep blue). The 3D surface plot (Fig. 8F) was drawn using ImageJ software to quantify the mean color intensities and numbers of these exposed axons, which corroborated that the loss of atg13 gene indeed impaired the myelin integrity in muscle-serving nerve fibers.

### Genetic depletion of ATG13 exacerbates deficits in energy metabolism and muscle fatigue following treadmill exercise.

**Figure 9:**
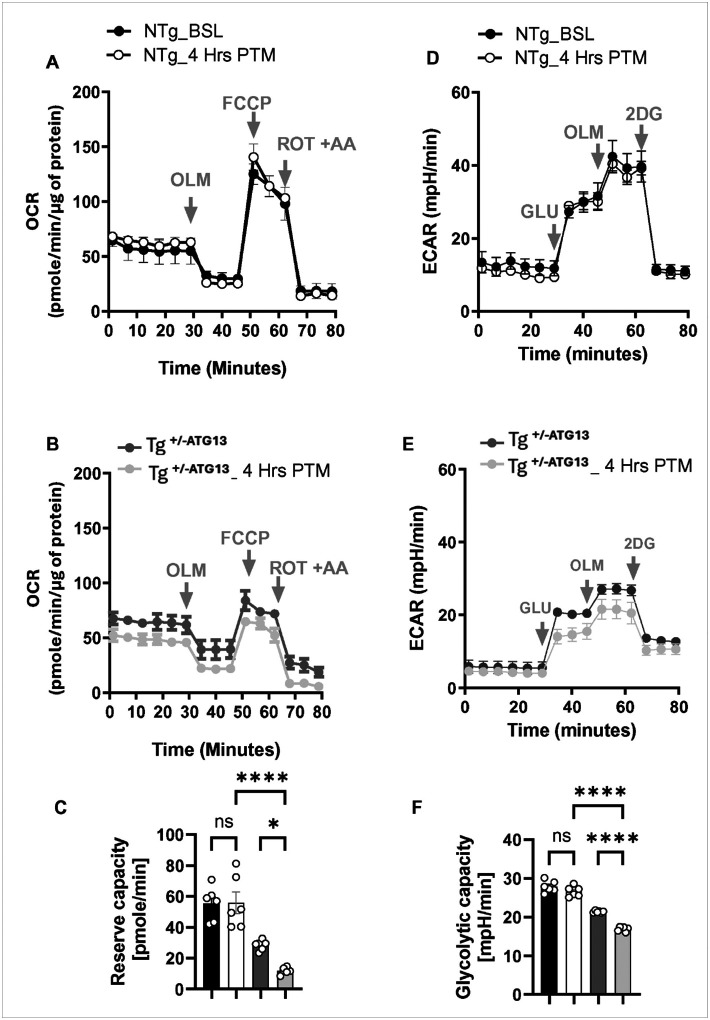
Seahorse metabolic analyses demonstrate that treadmill exercise exacerbates the impairments of mitochondrial energy metabolism and glycolysis in Tg^+/−ATG13^ mice. (A) Mitochondrial OCR study in purified splenic Mφ cells of NTg without (BSL =baseline) and after 4 hrs of post treadmill (PTM) (n=3/group). (B) Mitochondrial OCR study in splenic Mφs of Tg^+/−ATG13^ before and 4 hrs after treadmill exercise (n=3 per group). (C) spare or reserve capacity indicative of mitochondrial ability to restore OXPHOS after stress, was measured in all four groups. Black bars = NTg without treadmill, white bars = NTg after 4 hrs of treadmill, dark grey bars = Tg without treadmill, and lighter grey bars = Tg after 4 hrs of treadmill. Two-way ANOVA (effectors: genotype and stressor) was adopted, followed by multiple comparison analyses to monitor the significance of the mean between groups. ****p<0.0005, *p<0.05, and ns = no significance. Glycolysis was measured in enriched Mφs and then compared between (D) NTg without treadmill and 4 hrs PTM, as well as (E) Tg^+/−ATG13^ without treadmill and 4 hrs PTM (n=3 per group). (F) Glycolytic capacity, indicative of restorative ability after stress, was measured in all four groups. Two-way ANOVA was performed to test the significance of means between groups. ****p<0.0005, and ns = no significance. Results are mean ± SEM of three different experiments.

Next, we examined whether a stressor increases glycolytic lactic acid production (ECAR glycolytic capacity) and mitochondrial ATP production deficiency (OCR reserve capacity) in Tg^+/−ATG13^ mice. To induce stress, Tg^+/−ATG13^mice underwent 15-minute treadmill exercise at 14 rpm, rested for 4 hours, and then their splenic Mφ cells were isolated to measure mitochondrial OCR and glycolytic ECAR as described in the methods section. First, mitochondrial OCR assay (Fig. 9A) did not show any significant deficit in basal respiration, ATP-linked respiration or ATP turnover rate, and reserve capacity in NTg mice before and after treadmill exercise. Interestingly, after treadmill exercise, the OCR assay (Fig. 9B) assay revealed a significant deficit in basal respiration, ATP utilization and spare or reserve capacities. Quantitative assessment of reserve capacity, an indicator of OXPHOS activity under stress conditions, revealed a significant impairment (Fig. 9C) in splenic macrophages of Tg^+/−ATG13^ mice 4 hours following treadmill exercise. Next, we performed an ECAR assay to evaluate glycolysis. Similar to OCR assay, ECAR assay (Fig. 9D) revealed no change in glycolytic capacity under both basal and stressed conditions in NTg mice before and after exercise. However, there is a significant reduction of spare glycolytic capacity in Mφs of Tg+/−ATG13 mice (n = 3) 4 hours after exercise as shown in the last phase (post-2DG) of ECAR curve (Fig. 9E) followed by a histogram analysis (Fig. 9F). Collectively, these results indicate that atg13 depletion may exacerbate to the reduced mitochondrial ATP production and increased lactate production in Tg^+/−ATG13^mice following treadmill exercise.

**Figure 10:**
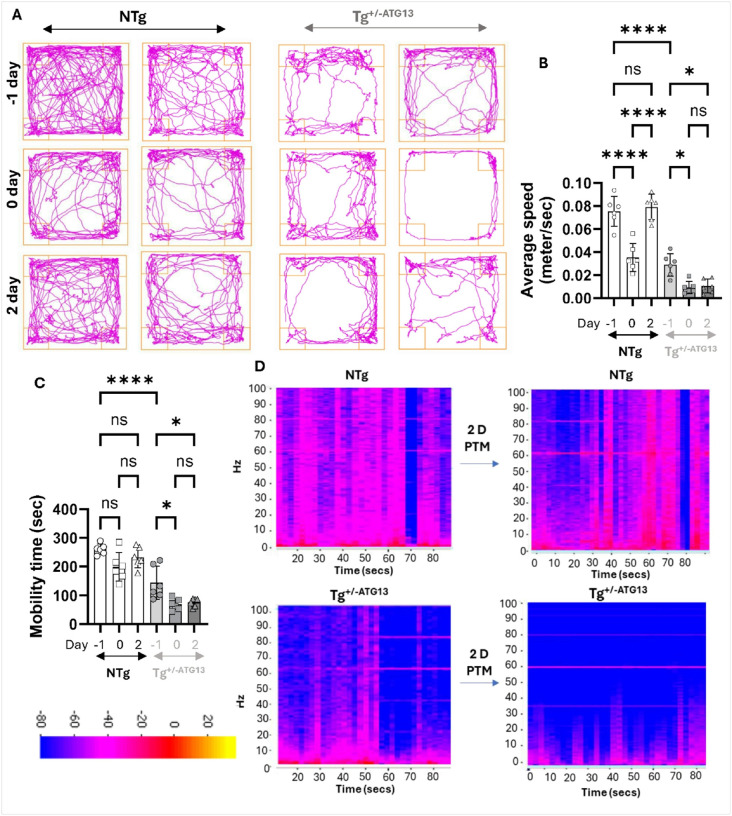
Measurement of muscle fatigue after treadmill exercise in Tg^+/−ATG13^ mice (A) Representative trackplots indicated the gross movement of both male (left) and female (right) NTg and Tg^+/−ATG13^ mice (10–12 weeks old) on the day before (−1 day), immediately after (0 day), and 2 days after treadmill exercise (4–14 rpm for 15 mins). (B) Average speed and (C) mobility time were calculated in different time points of treadmill exercise. Two-way Anova followed by Tukey’s multiple comparison analysis, resulted in the significance of mean between groups. ****p<0.0005 and *p<0.05. (D) EMG recording followed by summation of frequency was plotted as heatmap analysis. Results are mean ± SEM of three different experiments.

Next, we were interested to study if the post-treadmill exacerbations of mitochondrial and glycolytic impairments of energy metabolism contributed to the muscle fatigue in Tg^+/−ATG13^mice. The open-field behavioral studies (Fig. 10A) indicated that Tg^+/−ATG13^, not NTg mice, have severe movement deficits that worsens even after 2 days post- treadmill exercise. Quantitative estimations of average speed (Fig. 10B) and mobility time (Fig. 10C) further corroborated that the treadmill exercise indeed worsened the mobility of Tg^+/−ATG13, but^ not NTg mice, 2 days post-treadmill exercise. To understand that muscle weakness in molecular level, next we performed surface EMG recordings on biceps muscle in NTg and Tg^+/−ATG13^mice before and 2 days after treadmill exercise followed by a heatmap analysis (Fig. 10DD) to display summation of muscle wave frequencies. The summation of muscle waves were displayed in Hz scale for 1 minute. Interestingly, the EMG recording data in Tg^+/−ATG13^mice showed a severe loss of muscle frequencies at basal level that worsens 2 days post treadmill exercise, whereas no notable deficit was observed in NTg mice.

Overall, our results indicate that the depletion of atg13 followed by autophagy impairment significantly affected cellular redox metabolism through disruptions in cellular and mitochondrial energy metabolism, leading to inflammation and polarization of splenic macrophages into the M1 mode. Consequently, there is a strong perivascular infiltration of these inflammatory cells that results in a demyelinating response in muscle-serving nerve fibers.

## Discussion

ME/CFS[30, 31] is a chronic inflammatory disease[32] characterized by severe muscle fatigue[33] which worsens after physical or mental exhaustion[34]. The underlying molecular mechanism is still unknown. Our previous study indicated that the disruption of autophagy due to LacZ-mediated suppression of *atg13* gene induced severe muscle weakness, which was exacerbated after treadmill exercise. That observation intrigued us to study the molecular mechanism in detail. To pinpoint the molecular role of atg13 gene, we developed a universal cre-loxP conditional atg13 KO mice. Although homozygous KO mice are not viable, hemizygous KO mice (Tg^+/−ATG13^) are viable and displayed strong reduction of atg13 gene expression in spleen. Interestingly, these Tg^+/−ATG13^mice showed significantly enlarged and inflamed spleens with enhanced number of Mφ in the red pulp, suggesting the potential role of Mφ cells in systemic inflammation. The red pulp of the spleen is composed of sinusoids, which are open capillaries containing monocytes and macrophages. Resident Mφs in red pulp play crucial physiological roles in regulating antigenic responses and removing aged erythrocytes. Our histochemical studies indicated that genetic ablation of atg13 gene, increased the expression of IBA-1 in these Mφ cells, suggesting a direct role of *atg13* ablation in inducing an inflammatory phenotype in these cells. Further characterization of different surface proteins by flowcytometry confirmed that these activated Mφ cells acquire M1 inflammatory phenotype with strong expressions signature M1 surface proteins including CD40 and CD86. Similar flowcytometry analyses revealed that the acquisition of M1 properties also accompanies the downregulation of M2 surface proteins such as CD163 and CD206. While characterize the functional properties of these Mφ, our analyses revealed that these cells were associated with strong expressions of iNOS, activated NF-κB confirming the inflammatory M1 phenotype of these cells. Additionally, these cells lost phagocytic activity, decreased lysosomal function, and downregulated arginase, indicating the loss of M2Mφ function due to atg13 gene ablation. Consistent with our current finding, ours and other literatures also identified that the activation of NFκB, dysregulation of NO productions, and redox imbalance could play critical roles in the pathogenesis of ME/CFS. In ME/CFS, it is hypothesized that M1 macrophages, which are pro-inflammatory, could be relevant to the disease process. A study[35] suggested that classical monocytes in ME/CFS patients tend to migrate to tissues and become macrophages. High-resolution transcriptomics study after analyzing 13,000 transcriptomes in 33 ME/CFS patients followed by functional network analysis also identified upregulations of inflammatory mediators such as IL-8, NFκB, and TNF-α, associated with the M1Mφ function. Our previous study also demonstrated the activation of STAT3 in these inflammatory Mφs and downstream expressions of inflammatory cytokines such as IL6 and RANTES also play key role in the ME/CFS.

We studied the molecular mechanism of how atg13 gene ablation stimulated the polarization of M1 phenotype. The depletion of atg13 gene caused upscaled production of ROS suggesting that there may be dysregulation in cellular energy metabolism due to disrupted autophagy. Seahorse OCR analysis revealed that atg13 depletion significantly compromised the basal and stress-driven mitochondrial oxidative phosphorylation (OXPHOS) in Tg^+/−ATG13^Mφs. Interestingly, the Mitochondrial deficiency of OXPHOS worsened after treadmill exercise. On the other hand, the ECAR analysis indicated that atg13 inhibition also impaired the glycolytic flux, which is exacerbated after treadmill exercise. Given that extracellular acidification rate (ECAR) is a real-time indicator of overall lactate production [36], it is important to note that the absence of ATG13 may increase lactate production following stress. Elevated levels of inactivated phospho ATG13 have been previously reported [14] in the plasma samples of ME/CFS patients, indicating that the current findings are highly pertinent to the pathogenesis of ME/CFS. Moreover, the study of mitochondrial OXPHOS showed that ME/CFS patients have a deficiency in energy metabolism in immune cells, highlighting the relevance of our current research in the molecular mechanisms of disease progression.

Mitochondrial deficiency of energy metabolism, redox imbalance, and decreased glycolytic capacity contribute to our understanding of the polarization of macrophage (Mφ) cells to the M1 mode. However, the mechanism by which these metabolic deficits trigger an inflammatory response is not yet understood. Our current findings suggest that the increased nitrosative stress diminished the deacetylase enzymic activity of Sirtuin1 (SIRT1) causing the upregulation of acetylated fraction of activated NFκB. On contrary, SIRT2 activity was observed unaltered in spleen suggesting that diminished activity of SIRT1 may be responsible in stimulating the inflammation of splenic Mφs.

Our current manuscripts also highlights the potential mechanism of inflammation in skeletal muscle tissue with the augmented infiltration of these M1Mφ cells in the perivascular network of muscle parenchyma. Subsequent histological analysis identified that there as a significant impairment of myelin integrity in the muscle-serving nerve fibers of nerve spindles. EMG recording study also indicated that the weakening of skeletal muscle strength in these Tg^+/−ATG13^ mice, which exacerbated following treadmill exercise.

In summary, our research highlighted a novel mechanism of inflammation due to the depletion of atg13 gene followed by autophagy impairment that combines the mitochondrial impairment of energy metabolism, augmented ROS productions, upregulations of inflammatory markers, polarization of M1Mφs, enhanced infiltration into the muscle vasculature impairment of myelin integrity, and finally the chronic muscle weakness.

Future directions: We are currently conducting a decentralized observational clinical trial of mTOR inhibitor rapamycin in the alleviation of clinical symptoms of fatigue in ME/CFS. We are collecting plasma and PBMCs from the participating subjects before and 30, 60, and 90 days after 6mg/week doses of rapamycin. In the future course of study, we will conduct a M1/M2 polarization study in PBMC-derived Mφs. Also, we will investigate once applied if plasma-borne factors could polarize human Mφs to M1 phenotype.

## Figures and Tables

**Table 1.** The list of antibodies and application

Antibody (conjugate)	Catalogue#	Vendor	Application (dil^n^)
CD40	PA5-78980	Invitrogen	IB (1:500), IF (1:100)
LAMA5	PA5-49930	Invitrogen	IF (1:100)
ATG13	10181-2-AP	Protein tech	IB (1:500), IF (1:100)
ATG101	PA5-114272	Invitrogen	IB (1:200)
Beta-actin	PA5-85271 /AM4302	Invitrogen	IB (1:1000)
IBA1	MA5-50413	Invitrogen	IF (1:500)
S468P p65	500-11854	Amsbio	IF (1:250)
Rabbit LC3	14600-1-AP	Protein tech	IB, IF (1:250)
WDFY3	55009-1-AP	Protein tech	IHC (1:100), FACS (1:100)
3Nitrotyrosine	MA1-12770	Invitrogen	IF (1:100)
Acetyl p65	PA5-17264	Invitrogen	IF(1:100), IB (1:250)
SIRT1	MA5-30879	Invitrogen	IF (1:100); IB (1:250)
SIRT2	PA5-20487	Invitrogen	IB (1:500)
iNOS	18985-1-AP	Invitrogen	IF (1:100)
CD163(APC)	MA5-17717	Invitrogen	FACS (1:100)
CD11b (FITC)	11-0118-42	Invitrogen	FACS (1:100)
CD40 (APC)	17-0409-42	Invitrogen	FACS (1:100)
CD86 (PE)	12-0869-42	Invitrogen	FACS (1:100)
Arginase1 (APC)	17-3697-82	Invitrogen	FACS (1:100)
CD206 (APC)	17-2069-42	Invitrogen	FACS (1:100)

## Data Availability

There is no electronic datasheet associated with this paper. No data in an electronic repository.

## Highlights

1. The genetic deletion of atg13 gene induced the polarization and activation of M1Mφ cells in spleen.
2. The suppression of ATG13 induced mitochondrial deficit of oxygen consumption and augmented ROS production in splenic M1Mφ cells.
3. The deficit of oxygen metabolism in mitochondria inhibited cellular acetylase SIRT1 via nitrotyrosination and triggered inflammation via acetylation of p65.
4. The active infiltration of M1Mφ cells in the muscle vasculature contributed to the loss of myelin integrity in muscle-serving nerves.

## Abbreviations

ATG13: Autophagy related protein 13
Mφ: Macrophage
Tg^+/−ATG13^: Hemizygous deletion of ATG13 transgenic mouse
CD: Cluster of differentiation
ME/CFS: Myalgic Encephalomyelitis/ Chronic Fatigue Syndrome
PEM: post-exertional malaise
ROS: Reactive oxygen species
IF: Immunofluorescence
IB: immunoblot
FACS: Flow-cytometry Activated Cell Sorting
OCR: Oxygen consumption rate
ECAR: Extracellular acidification rate

## References

[1] Mizushima N. Autophagy: process and function. Genes & development 2007; 21:2861–2873. 18006683 10.1101/gad.1599207

[2] Dunn WA Jr. Autophagy and related mechanisms of lysosome-mediated protein degradation. Trends in cell biology 1994; 4:139–143. 14731737 10.1016/0962-8924(94)90069-8

[3] Zhang J. Autophagy and mitophagy in cellular damage control. Redox biology 2013; 1:19–23. 23946931 10.1016/j.redox.2012.11.008 PMC3740586

[4] Xie Z, Klionsky DJ. Autophagosome formation: core machinery and adaptations. Nature cell biology 2007; 9:1102–1109. 17909521 10.1038/ncb1007-1102

[5] Mizushima N. The ATG conjugation systems in autophagy. Current opinion in cell biology 2020; 63:1–10. 31901645 10.1016/j.ceb.2019.12.001

[6] Cao QH, Liu F, Yang ZL, Fu XH, Yang ZH, Liu Q, Prognostic value of autophagy related proteins ULK1, Beclin 1, ATG3, ATG5, ATG7, ATG9, ATG10, ATG12, LC3B and p62/SQSTM1 in gastric cancer. Am J Transl Res 2016; 8:3831–3847. 27725863 PMC5040681

[7] Inoue Y, Suzuki T, Hattori M, Yoshimoto K, Ohsumi Y, Moriyasu Y. AtATG genes, homologs of yeast autophagy genes, are involved in constitutive autophagy in Arabidopsis root tip cells. Plant and Cell Physiology 2006; 47:1641–1652. 17085765 10.1093/pcp/pcl031

[8] Li W, Zhang L. Regulation of ATG and autophagy initiation. Autophagy: Biology and Diseases: Basic Science: Springer, 2019:41–65. 10.1007/978-981-15-0602-4_2 31776979

[9] Puente C, Hendrickson RC, Jiang X. Nutrient-regulated phosphorylation of ATG13 inhibits starvation-induced autophagy. Journal of Biological Chemistry 2016; 291:6026–6035. 26801615 10.1074/jbc.M115.689646 PMC4786734

[10] Barth J, Szabad J, Hafen E, Köhler K. Autophagy in Drosophila ovaries is induced by starvation and is required for oogenesis. Cell Death & Differentiation 2011; 18:915–924. 21151027 10.1038/cdd.2010.157 PMC3131947

[11] Suttangkakul A, Li F, Chung T, Vierstra RD. The ATG1/ATG13 protein kinase complex is both a regulator and a target of autophagic recycling in Arabidopsis. The Plant Cell 2011; 23:3761–3779. 21984698 10.1105/tpc.111.090993 PMC3229148

[12] Pan L, Liu J, Li Y. Structural basis of autophagy regulatory proteins. Autophagy: Biology and Diseases: Basic Science 2019:287–326. 10.1007/978-981-15-0602-4_15 31776992

[13] Drosen ME, Bulbule S, Gottschalk G, Peterson D, Allen LA, Arnold LA, Inactivation of ATG13 stimulates chronic demyelinating pathologies in muscle-serving nerves and spinal cord. Immunologic Research 2025; 73:27. 39777574 10.1007/s12026-024-09557-7 PMC11706859

[14] Gottschalk G, Peterson D, Knox K, Maynard M, Whelan RJ, Roy A. Elevated ATG13 in serum of patients with ME/CFS stimulates oxidative stress response in microglial cells via activation of receptor for advanced glycation end products (RAGE). Molecular and Cellular Neuroscience 2022; 120:103731. 35487443 10.1016/j.mcn.2022.103731

[15] Davenport TE, Chu L, Stevens SR, Stevens J, Snell CR, Van Ness JM. Two symptoms can accurately identify post-exertional malaise in myalgic encephalomyelitis/chronic fatigue syndrome. Work 2023; 74:1199–1213. 36938769 10.3233/WOR-220554

[16] UK NGC. Identifying and diagnosing ME/CFS. 2021.

[17] Fluge Ø, Mella O, Bruland O, Risa K, Dyrstad SE, Alme K, Metabolic profiling indicates impaired pyruvate dehydrogenase function in myalgic encephalopathy/chronic fatigue syndrome. JCI insight 2016; 1:e89376. 28018972 10.1172/jci.insight.89376 PMC5161229

[18] Booth NE, Myhill S, McLaren-Howard J. Mitochondrial dysfunction and the pathophysiology of myalgic encephalomyelitis/chronic fatigue syndrome (ME/CFS). International journal of clinical and experimental medicine 2012; 5:208. 22837795 PMC3403556

[19] Tomas C, Elson JL, Strassheim V, Newton JL, Walker M. The effect of myalgic encephalomyelitis/chronic fatigue syndrome (ME/CFS) severity on cellular bioenergetic function. PLoS One 2020; 15:e0231136. 32275686 10.1371/journal.pone.0231136 PMC7147788

[20] Mandarano AH, Maya J, Giloteaux L, Peterson DL, Maynard M, Gottschalk CG, Myalgic encephalomyelitis/chronic fatigue syndrome patients exhibit altered T cell metabolism and cytokine associations. The Journal of clinical investigation 2020; 130:1491–1505. 31830003 10.1172/JCI132185 PMC7269566

[21] Radbruch A. Immunofluorescence: basic considerations. Flow cytometry and cell sorting: Springer, 2000:38–52.

[22] Villalta SA, Nguyen HX, Deng B, Gotoh T, Tidball JG. Shifts in macrophage phenotypes and macrophage competition for arginine metabolism affect the severity of muscle pathology in muscular dystrophy. Human molecular genetics 2009; 18:482–496. 18996917 10.1093/hmg/ddn376 PMC2638796

[23] Alanazi FJ, Alruwaili AN, Aldhafeeri NA, Ballal S, Sharma R, Debnath S, Pathological interplay of NF-κB and M1 macrophages in chronic inflammatory lung diseases. Pathology-Research and Practice 2025:155903. 40081284 10.1016/j.prp.2025.155903

[24] Stujanna EN, Murakoshi N, Tajiri K, Xu D, Kimura T, Qin R, Rev-erb agonist improves adverse cardiac remodeling and survival in myocardial infarction through an anti-inflammatory mechanism. PloS one 2017; 12:e0189330. 29232411 10.1371/journal.pone.0189330 PMC5726719

[25] Suuronen T, Huuskonen J, Nuutinen T, Salminen A. Characterization of the pro-inflammatory signaling induced by protein acetylation in microglia. Neurochemistry international 2006; 49:610–618. 16797784 10.1016/j.neuint.2006.05.001

[26] Desousa BR, Kim KK, Jones AE, Ball AB, Hsieh WY, Swain P, Calculation of ATP production rates using the Seahorse XF Analyzer. EMBO Rep 2023; 24:e56380. 37548091 10.15252/embr.202256380 PMC10561364

[27] He S, Wang Y, Liu J, Li P, Luo X, Zhang B. Activating SIRT1 deacetylates NF-κB p65 to alleviate liver inflammation and fibrosis via inhibiting NLRP3 pathway in macrophages. Int J Med Sci 2023; 20:505–519. 37057212 10.7150/ijms.77955 PMC10087625

[28] Rothgiesser KM, Erener S, Waibel S, Lüscher B, Hottiger MO. SIRT2 regulates NF-κB dependent gene expression through deacetylation of p65 Lys310. J Cell Sci 2010; 123:4251–8. 21081649 10.1242/jcs.073783

[29] He S, Wang Y, Liu J, Li P, Luo X, Zhang B. Activating SIRT1 deacetylates NF-κB p65 to alleviate liver inflammation and fibrosis via inhibiting NLRP3 pathway in macrophages. International Journal of Medical Sciences 2023; 20:505. 37057212 10.7150/ijms.77955 PMC10087625

[30] Carruthers BM, Jain AK, De Meirleir KL, Peterson DL, Klimas NG, Lerner AM, Myalgic Encephalomyelitis/Chronic Fatigue Syndrome. Journal Of Chronic Fatigue Syndrome 2003; 11:7–115.

[31] Jason LA, Fennell PA, Taylor RR. Handbook of chronic fatigue syndrome: John Wiley and Sons New York, 2003.

[32] Jonsjö MA, Olsson GL, Wicksell RK, Alving K, Holmström L, Andreasson A. The role of low-grade inflammation in ME/CFS (Myalgic Encephalomyelitis/Chronic Fatigue Syndrome)-associations with symptoms. Psychoneuroendocrinology 2020; 113:104578. 31901625 10.1016/j.psyneuen.2019.104578

[33] Gerwyn M, Maes M. Mechanisms explaining muscle fatigue and muscle pain in patients with myalgic encephalomyelitis/chronic fatigue syndrome (ME/CFS): a review of recent findings. Current rheumatology reports 2017; 19:1. 28116577 10.1007/s11926-017-0628-x

[34] Loy BD, O’Connor PJ, Dishman RK. Effect of acute exercise on fatigue in people with ME/CFS/SEID: a meta-analysis. Medicine and science in sports and exercise 2016; 48:2003. 27187093 10.1249/MSS.0000000000000990 PMC5026555

[35] Vu LT, Ahmed F, Zhu H, Iu DSH, Fogarty EA, Kwak Y, Single-cell transcriptomics of the immune system in ME/CFS at baseline and following symptom provocation. Cell Reports Medicine 2024; 5:101373. 38232699 10.1016/j.xcrm.2023.101373 PMC10829790

[36] Legmann R, Melito J, Belzer I, Ferrick D. Analysis of glycolytic flux as a rapid screen to identify low lactate producing CHO cell lines with desirable monoclonal antibody yield and glycan profile. BMC Proc 2011; 5 Suppl 8:P94. 22373163 10.1186/1753-6561-5-S8-P94 PMC3284893

